# A comparative study of non-covalent encapsulation methods for organic dyes into silica nanoparticles

**DOI:** 10.1186/1556-276X-6-328

**Published:** 2011-04-13

**Authors:** Aurélien Auger, Jorice Samuel, Olivier Poncelet, Olivier Raccurt

**Affiliations:** 1 CEA Grenoble, Department of Nano Materials, NanoChemistry and NanoSafety Laboratory (DRT/LITEN/DTNM/LCSN), 17 rue des Martyrs, 38054 Grenoble Cedex 9, France

## Abstract

Numerous luminophores may be encapsulated into silica nanoparticles (< 100 nm) using the reverse microemulsion process. Nevertheless, the behaviour and effect of such luminescent molecules appear to have been much less studied and may possibly prevent the encapsulation process from occurring. Such nanospheres represent attractive nanoplatforms for the development of biotargeted biocompatible luminescent tracers. Physical and chemical properties of the encapsulated molecules may be affected by the nanomatrix. This study examines the synthesis of different types of dispersed silica nanoparticles, the ability of the selected luminophores towards incorporation into the silica matrix of those nanoobjects as well as the photophysical properties of the produced dye-doped silica nanoparticles. The nanoparticles present mean diameters between 40 and 60 nm as shown by TEM analysis. Mainly, the photophysical characteristics of the dyes are retained upon their encapsulation into the silica matrix, leading to fluorescent silica nanoparticles. This feature article surveys recent research progress on the fabrication strategies of these dye-doped silica nanoparticles.

## Introduction

The development and need for silica-based fluorescent nanoparticles as markers in biological applications such as sensing and imaging have spread significantly since the 1990s [1-3]. Fluorescent labelling of biomolecules has been established as an essential tool in many biological investigations. Recently, significant advances have led to a large variety of labelling reagents based on inorganic (quantum dots [4], lanthanide-doped oxides [5,6], metallic gold [7,8]) or organic nanomaterials (latex, polystyrene and polymethylmethacrylate) [9]. Indeed, small luminescent molecules like organic dyes displaying high quantum yield can be encapsulated into oxide nanoparticles, specifically into silica, by sol-gel. These new fluorescent probes can be developed for the field of biological assays and have reached great expectations [10,11]. The wide range and variety of fluorophores available nowadays facilitate the targeting of suitable applications for the newly prepared nanoparticle materials.

Organics dyes have been known for some time now to be used in biology for fluorescent labelling. Although those dyes possess a certain number of drawbacks including a short Stokes shift, poor photochemical stability, sensibility to the buffer composition (quenching or decomposition due to the pH), susceptibility to photobleaching and decomposition under repeated excitation, they remain used extensively and considerably as a result of their low cost, commercial availability and ease of use. Furthermore, modern research has developed organic dyes which exhibit better chemical and optical properties. Examples involve fluorescein [12,13], rhodamine [14,15], cyanine [13,16], alexa dyes [13,17], oxazines [18,19], porphyrins [20] and phthalocyanines [21], just to name a few. Even if fluorescence detection exhibits a sharp sensitivity, most of the organic fluorophores used as luminescent biomarkers present drawbacks. Therefore, hydrophobicity (causing a poor solubility into biological buffers) (collisional), quenching in aqueous media and irreversible photodegradation under intense excitation light [11,22], requires encapsulation so that to produce monodisperse and more robust emitters from organic dye molecules and amorphous silica. Furthermore, a supplementary advantage to encapsulation of organic dyes into silica beads is to enhance the detection limit by encapsulating a larger number of fluorophores molecules by synthesised probes. The technique of encapsulation of fluorophores into silica beads prevents from interaction of fluorophores with the buffer. Finally, silica functionalisation is a well-known and a well-developed chemistry, and the incorporation of dyes into silica nanoparticles offer a great potential for customising the surface independently to the dye structure.

Traditionally, there are two chemical approaches for incorporating organic dyes into silica nanoparticles. The first approach consists of using covalent bonding of the dye with the silicated matrix [23-25]. On the contrary, the second approach has been described as using non-covalent or non-bonding process (i.e. by electrostatic interactions), by entrapping the dye into the siloxane matrix [26]. Relatively few examples (involving rhodamine and ruthenium complexes) have been reported in the literature, and covalent binding of the dye to the silica network is usually the preferred method. Sol-gel synthesis of silica beads can also be undertaken by two types of sol-gel methods: the Stöber [27] and the microemulsion methods [28]. It is obvious that the best method for incorporation of a dye into silica beads is by the covalent bonding approach but it requires the dye to possess sufficient chemical groups towards functionalisation and chemical reaction between the dye and the silicated precursors. This concept might sometimes enhance considerably the difficulty of the dye preparation. Consequently, the non-covalent approach represents a promising way and more attention should be paid to its investigation since it exhibits a low-cost method, and that this process does not emphasise the limitation of the chosen dye.

According to the Stöber method, the incorporation yield of the dye into the silica beads under non-covalent bonding is poor and dependant of the absorption force between the dye itself and the silica precursor [15]. However, the microemulsion process avoids that drawback, controlling the quantity of incorporated dye into silica beads by utilising a water soluble dye. For reminding, the first method has been developed in the late 1960s by Stöber et al. [27]. The mild synthetic protocol consists of the hydrolysis and condensation of silica alkoxide precursors (such as tetraethoxysilane, TEOS) in ethanol solution in the presence of aqueous ammonium hydroxide mixture as a catalyst to generate electrostatically stabilised, spherical and monodisperse particles. Indeed, homogeneous nucleation forms silica particles of tens to hundreds of nanometres in size [28,29]. Even if this method is rather simple and that it can involve the incorporation of both organic and inorganic markers [19], the fact is that the particle size may not be uniform and besides different modifications of the particle surface are not easily achieved and might require covalent binding to achieve proper encapsulation. The second approach for the synthesis of uniform organic dye-doped silica nanoparticles of different sizes can be achieved by a reverse microemulsion method [30-33]. Reverse microemulsion techniques rely on the stabilisation of water nanodroplets (by surfactant molecules) formed in an oil solution (water in oil (W/O) emulsion) which act as nanoreactors, where silane derivatives hydrolysis and formation of nanoparticles take place, entrapping dye molecules [11,26]. Furthermore, the nanoreactor environment within the reverse micelle has been yielded highly monodisperse nanoparticles and an increase in the incorporation of nonpolar molecules has been observed [34] because the particle's dimension was limited by the volume of the micelle. The microemulsion method produced hydrophilic and fairly uniform-sized nanoparticles and allows easy modulation of the nanoparticle surfaces for various applications. Moreover, it has been determined that the size of the nanoparticles is controlled by parameters such as the hydrolysis reagent, the nature of surfactant, the reaction time and the oil/water ratio, just to name a few [28].

Dye encapsulation can be achieved either by covalent bond of the dye with silica precursors before the hydrolysis or by first solubilising the dye in the core (small reactors) of the microemulsion and then carrying out the polymerisation. As a matter of fact, the covalently dye-doped silica nanoparticles have launched a promising field towards the development and investigation of luminescent biomarkers. Many studies on this topic were reported [11,28,30-32], principally since 1992, van Blaaderen and co-workers [23,24] described for the first time covalently incorporating organic fluorophores into the silica matrix by coupling them to reactive organosilicates. This approach affords versatility with regard to the placement of the dye molecules within the silica nanoparticle. The non-covalent approach has recently been subjected to investigation by Tan and co-workers, who reported that fluorophores (e.g. rhodamine 6G) can be captured at high concentrations in silica nanoparticle cores produced by means of a reverse microemulsion process [34-36]. The water-soluble fluorophores are confined in the polar core of the inverse micelles in which hydrolysis as well as nanoparticle formation take place, leading to the dye incorporation into the sol-gel matrix of the nanoparticles [37].

Encapsulation of hydrophobic molecules by reverse microemulsion has also been investigated [15]. Further to their study, Deng et al. [38] described the use of a silica precursor, hexadecyltrimethoxysilane (HDTMOS), mixed with a hydrophobic fluorophores, methylene blue (MB). This mixture, once added to TEOS, allowed the hydrophobic dye to be dragged in the silica nanoparticles during the synthetic process. The ratio of HDTMOS/MD and the synthetic procedure have been optimised to measure the incorporation rate of the dye by means of fluorescence spectroscopy. However, the lack of covalent connection between the fluorophores and the silica core imply that the dye molecules can leak out of the nanoparticles over time, inducing reduction of brightness of the material, amplification of background signal and exposition of the fluorophores to their environment.

Different requirements should characterise those nanoparticles to achieve the desired properties. Therefore, photostability, brightness as well as monodispersivity of the synthesised nanoparticles should be targeted and focussed on. To the best of our knowledge, most of the reports concentrated on the incorporation of dyes or fluorophores through covalent bonds into colloidal silica spheres [39-43], which can greatly decrease the leakage from the silica matrix. Nevertheless very few studies have been carried out that focus on the nature of the fluorophores used for encapsulation and their effects either on the efficiency of the loading or the leaching of the dye-doped nanoparticles in a systematic manner. A major understanding of these phenomenons will provide the elemental basis for the effective application of these silica nanoparticles in the topics of bioanalysis and bioseparation. In this study, we report the effect of the nature of the fluorophores molecules on the particle size, polydispersity, loading and fluorescence spectra of dye-doped silica nanoparticles produced by the reverse microemulsion sol-gel synthesis.

## Materials and methods

### Materials

Triton^® ^X100 (TX-100), 1-hexanol anhydrous (≥99%), cyclohexane reagent plus^® ^(≥99%), aqueous ammonia (NH_4_OH) solution (25%), tetramethylorthosilicate (TMOS, 98%), tetraethylorthosilicate (TEOS, 98%), ethanol, Cardiogreen (ICG), Fluorescein, Rhodamine B, Propyl Astra Blue Iodide (PABI), 4,4',4",4'''-(Porphine-5,10,15,20-tetrayl)tetrakis(benzoic acid) (PPC), IR 806, Nile Blue A perchlorate (NBA), 1,1',3,3,3',3'-Hexamethylindotricarbocyanine iodide (HITC), all purchased from Aldrich, were used without further purification. Water was purified with a Milli-Q system (Millipore, Bedford, MA, USA) including a SynergyPak^® ^unit. The exclusive Jetpore^®^, ultrapure grade mixed-bed ion-exchange resin, was also used in this unit. Water achieved resistivity above 18.0 MΩ · cm at 25°C. A C 3.12 centrifuge (Jouan, France) and a SONOREX DIGITEC sonification water-bath (Roth, France) were used.

### Synthesis

#### General method of dye encapsulation

Silica nanoparticles were synthesised using a reverse microemulsion method, as described by Bagwe et al. [28] in the literature. Consequently, a quaternary microemulsion consisted of mixing Triton X-100 (4.2 ml), 1-hexanol (4.1 ml) and cyclohexane (18.76 ml) under a vigorous stirring at room temperature, followed by additions of a concentrated aqueous solution of the selected dye in water (200 μL at 0.1 M), water (1.00 mL), aqueous ammonia NH_4_OH (250 μL at 25%) and TEOS (250 μL) or TMOS (250 μL) in that order. The mixture was allowed to stir for 24 h at room temperature and a subsequent addition of ethanol (100 mL) disrupted the inverse micelles. Particles were recovered by centrifugation (6000 × *g *for 15 min) and washed thoroughly three time with ethanol and once with water. Ultrasonification was used to disperse nanoparticles aggregated into the washing solvent and to increase the desorption rate of surfactant from the surface of the synthesised nanoparticles.

#### Capping of silica nanoparticles

Capping was achieved by adding TMOS (25 μL) to the reverse micellar system prior to disruption with ethanol. After stirring for 24 h at room temperature, the colloidal solution was subjected to a thermal treatment (30 min at 70°C), before separating and washing the so-formed capped silica nanoparticles with ethanol and water as in the procedure described above.

### Characterisation: transmission electron microscopy (TEM)

The morphology and sizes of dye-doped silica nanoparticles were obtained utilising a transmission electron microscope (JEOL 2000 FX). The sample for TEM was prepared by plunging a 200 mesh carbon-coated copper grid, 30-50 nm thickness (Euromedex, France) in the desired nanoparticle-containing aqueous solution just after dispersion by ultrasonification. Further to the evaporation of the water, the particles were observed at an operating voltage of 200 kV. Once the samples were imaged, TEM micrographs of dye-doped silica nanoparticles were converted to digitised images using imaging software (IMIX, PGT). Furthermore, elemental analysis of the samples could be performed by energy dispersion RX spectroscopy (EDS).

### Particle sizing

The hydrodynamic diameter and dispersivity of the silica nanoparticles were determined by dynamic light scattering (DLS) Technique using a Zetasizer Nano ZS from Malvern Instruments. The light scattering measurements were performed using a 633-nm red laser in a back-scattering geometry (*θ *= 180°). The particle size was analysed using a dilute suspension of particles in deionized (or ultrapure) water.

### Fluorescence measurements

All fluorescence measurements were performed at room temperature on a steady-state FS920 spectrofluorimeter (Edinburgh Instruments, UK,, Edinburgh, ) with a high spectral resolution (signal to noise ratio > 6000:1), using water as the solvent, and either a 1-cm cell or a 1-mm quartz cell, the latter oriented at -45° to the direction of the excitation light beam. The spectrofluorimeter covers the wavelength range from 200 to 1670 nm using two detectors: a photomultiplier R928 for UV-Vis scans (up to 870 nm) and a solid InGas TE G8605-23 detector for IR scans. The excitation source is a continuous Xenon Arc lamp (450 W) coupled to two Czerny-Turner DMX300X 1800 tr/mn monochromators, one for UV excitation (focal length 300 nm) and one for visible wavelength (focal length 500 nm). Fluorescence intensity values were integrated over the wavelength region specified. Data were recorded in a comparative manner, causing the same aperture of slits.

Transmission measurements were also recorded on a steady-state FS920 spectrofluorimeter (Edinburgh Instruments, UK) equipped with a Si solid detector and covering the wavelength range from 200 to 900 nm. For each sample, the reference spectrum of transmission was measured with the pure solvent (deionised water), and was subtracted from each sample transmission spectrum. Measurements were realised using 1 cm × 1 cm quartz cells.

## Results and discussion

### Preparation of dye-doped nanoparticle dispersions

**Figure 1:**
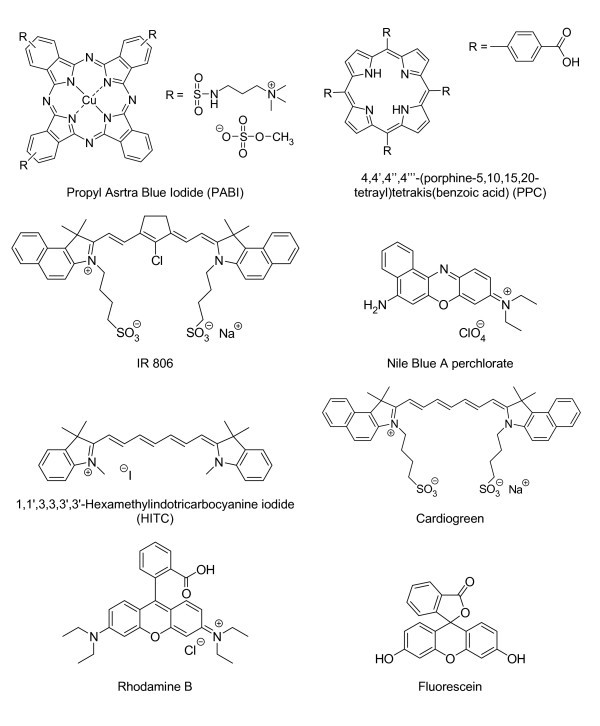
**Names and structures of the different dyes and fluorophores used during the study**.

We have synthesised luminescent probes based on silica nanoparticles embedded with different hydrophilic and organic dyes (Figure 1). The criteria and the parameters required to properly encapsulating those fluorophores within the silica shell have been investigated and seem to differ from one fluorophore to another. The success of relatively good encapsulation tends to be related to the structure of the selected dye. The first series of silica nanoparticles, 1a-h, was prepared using the recently developed W/O microemulsion method proposed by Bagwe et al. [28] This regular synthesis involved the use of Triton X100, *n*-hexanol, cyclohexane and water to prepare the microemulsion. The desired dye (Rhodamine B, Fluorescein, PABI, PPC, IR 806, NBA, HITC, ICG see Figure 1 for full names and structures) was dissolved in the aqueous phase at a concentration of 0.1 M in 200 μl, and injected in the W/O microemulsion system. The second step involves the hydrolysis of TEOS initiated by the addition of aqueous ammonia to the reaction mixture that results in the formation of monodisperse spherical particles of amorphous silica.

The second series of silica nanoparticles, 2a-f, was prepared in an identical way but using another silica precursor tetramethoxysilane (TMOS) for further capping of the produced nanoparticles, this second silica precursor was added after 24 h of reaction into the microemulsion to create a denser silica shell. A thermal treatment was effected at the end of the process so that to densify the silica network. This protocol was developed to investigate if the capping followed by a thermal treatment would re-enforce the encapsulation process and therefore behave more efficiently towards the encapsulation phenomenon. Indeed, it is known that the use of TMOS instead of TEOS produces a denser silica network, emphasising the encapsulation of the selected fluorophores. The use of TMOS is expecting to consolidate the silica shell of the produced materials by generating a denser silica network within the nanomaterials as suggested for the capping of the series 2. In addition, it is known from the literature that using standard conditions, the rate of hydrolysis of TEOS to a gel is about 10 days, whereas those of TMOS and tetra-*n*-butoxysilane (TBOS) are 2 and 25 days, respectively [44-47].

The third series of silica nanoparticles, 3a-f, was produced by mixing porous silica nanoparticles, which pores were functionalised with 3-(mercaptopropyl)triethoxysilane [48], with the proper aqueous solution of the required fluorophore. The thiol functionalities are design to bind and therefore trap the fluorophores within the pores of the silica nanoparticles. Finally, the fourth series of silica nanoparticles, 4a-f, was prepared exactly as the first series, 1, except that the silicon derivative used for hydrolysis was TMOS [49]. Further to washings four silica nanoparticles series (1-4) were isolated which physical properties were further investigated.

### Characterisation of nanoparticles

**Figure 2:**
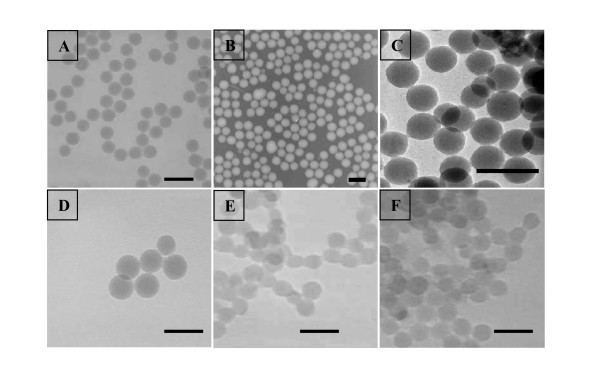
**TEM images of silica nanoparticles with different average sizes**. **(A) **1b (44 ± 3 nm), **(B) **2b (46 ± 3 nm), **(C) **1 h (46 ± 3 nm), **(D) **2b (47 ± 4 nm), **(E) **4b (40 ± 3 nm) and **(F) **4a (41 ± 4 nm). Scale bar: 100 nm.

**Figure 3:**
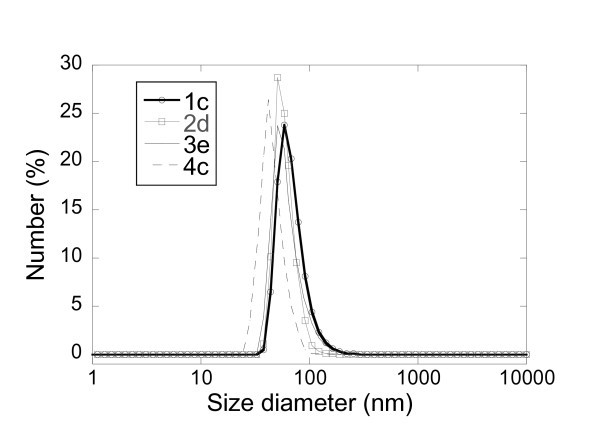
**Dynamic light scattering measurements of synthesized dye-doped silica nanoparticles of each series (1c 58 nm, 2d 50 nm, 3e 51 nm and 4c 44 nm)**.

Figure 2 shows TEM images of three different series (1, 2 and 4) of silica nanoparticles prepared in this study. No example illustrates the series 3. Indeed, due to the porosity of the material obtained at the extremely low pressure required for TEM analysis, the sample was (collapsed) crushed on itself and the pictures observed were not characteristic of the material. Cryo-TEM analysis of the material is under investigation in our laboratories and will be reported in a different manuscript. Overall, the resulting luminescent probes are spherical in shape, and average diameters of 44 ± 3, 47 ± 4 and 41 ± 4 nm have been observed for samples of each series ca. 1b, 2b and 4a, respectively. The images also showed that the particles were monodispersed. Further TEM images of samples 1g 48 ± 4 nm and 1 h 46 ± 3 nm are also available in Figure 2 so that to emphasise the size homogeneity obtained for different samples of the series 1. Dynamic laser light scattering measurements show that the hydrodynamic diameters (the apparent diameter of the hydrated/solvated particles) of each particle of each series (1-4) are slightly larger than the dry particle diameters observed from the TEM. The hydrodynamic diameters of the luminescent nanoparticles may be considerably larger than their 'dry' diameters due to the existence of a water layer surrounding the hydrophilic silica network. Therefore the following diameters of 58, 50, 51 and 44 nm were recorded for samples of each series, ca. 1c, 2d, 3e and 4c, respectively, as illustrated in Figure 3. Overall the TEM and DLS analyses have confirmed similar sizes, morphologies and dispersivity of the silica nanoparticles prepared using the different protocols.

### Spectroscopic properties of aqueous photoresponsive nanoparticle dispersions

The principal tools used in this study to characterise the dye's encapsulation into silica matrix are the absorption and fluorescence spectroscopies. The photochromic properties displayed by the nanoparticles are indicative of the successful incorporation of dyes into the nanoparticles. Indeed, to detect the correct encapsulation of the desired dye within the silica network of the nanoparticles, the fluorescence and/or the absorption of the aqueous solution of the prepared nanoparticles was measured. Such measurements informed us of the successful encapsulation. The following dyes have been subjected to encapsulation by four different methods described in the paragraph above.

Fluorescence and absorption measurements of every sample were recorded, and when a specific sample of nanoparticles exhibited such properties, it was immediately compared to the fluorescence or the absorption of the free-dye dissolved in water. References spectra of the different dyes in water had to be recorded so that to be able to compare the fluorescence recorded of the different fluorophores alone and also the fluorescence recorded of the fluorophores once encapsulated into the silica matrix.

The content or concentration of fluorescent dye in silica nanoparticles tends to influence the fluorescence intensity of nanoparticles dispersions. The quantity of encapsulated dye is not relevant to our study. Therefore, since the study mainly focuses on the incorporation and not the quantity of dyes into the silica matrix, all absorption and fluorescent spectra were normalised arbitrarily. Furthermore, self-quenching of fluorescence has been determined for each fluorophores used to establish the appropriate amount of chromophore to incorporate into the nanoparticles to ensure high fluorescence intensity and at the same time to avoid fluorescence self-quenching. The dyes selected for the study were: PABI, PPC, IR 806, NBA, HITC and ICG (Figure 1). We also reproduced the encapsulation of fluorescein and rhodamine with the standard microemulsion sol-gel process as the successful encapsulation of those two dyes has been investigated and optimised in our laboratories [50]. It is important to mention that all dyes and fluorophores selected for this study are commercially available and their hydrophilic structural character confer them good to excellent water solubility. High concentration such as 0.1 M in water was therefore employed for the synthetic processes 1-4.

#### Fluorescein and rhodamine

**Figure 4:**
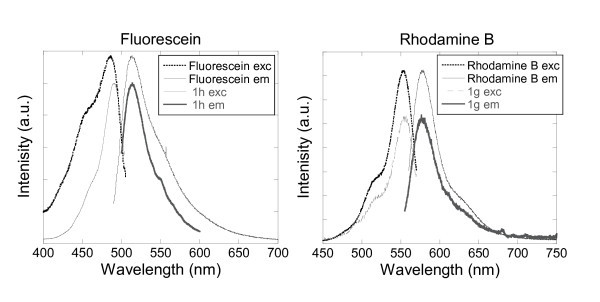
**Excitation and emission spectra of aqueous solutions of (left) fluorescein and silica nanoparticles doped with fluorescein 1 h, and (right) rhodamine B and silica nanoparticles doped with rhodamine B 1g**.

Furthermore, fluorescein and rhodamine B were successfully encapsulated by the method 1. The fluorescence data, excitation and emission wavelengths, observed for sample 1 h were identical to those recorded for a solution of free fluorescein in water as illustrated in Figure 4. Indeed, the fluorescence maximum, at 513 nm upon an excitation at 488 nm for sample 1 h, indicated that the fluorescein had been encapsulated into the silica nanoparticles. A freshly prepared solution of fluorescein into water also exhibited maxima excitation and emission wavelengths at 486 and 513 nm, respectively. The same phenomena were observed for the sample 1g consisting of rhodamine B encapsulated into silica nanoparticles. Both, the aqueous solutions of free rhodamine B and of sample 1g displayed maxima excitation and emission wavelengths at 555 and 577 nm, respectively. A slight shift and different shapes in the excitation band of the fluorescein was observed which is attributed to the incorporation of the fluorescent dye and its interaction with the silica network. Those results indicated that the silica encapsulation by microemulsion was suitable for encapsulation of hydrophilic chromophores and was consistent with the literature [15,51-54]. It was therefore decided after those fluorescence measurements (Figure 4) that no better encapsulation could be achieved by other processes and no further investigation of those two dyes were tested. It was also important to notice that non-covalent encapsulation of those dyes has been reported earlier on in the literature [52].

#### 3.3.2. PABI

**Figure 5:**
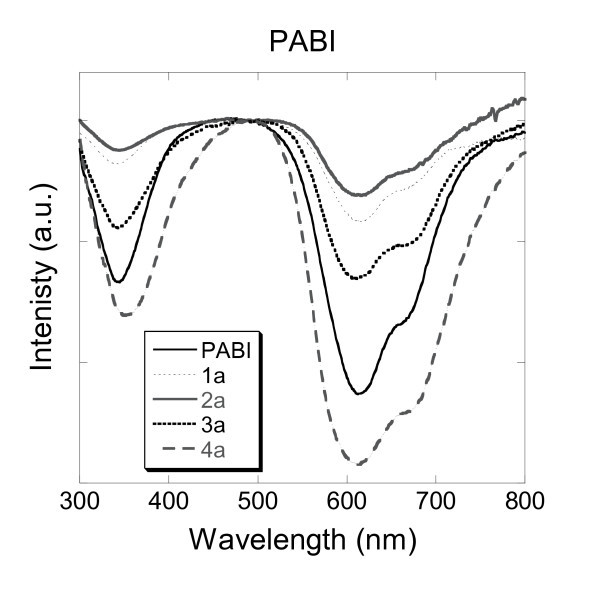
**Transmittance spectra of aqueous solutions of PABI and silica nanoparticles doped with PABI (1a, 2a, 3a and 4a)**.

The transmission spectra of pure PABI dye and PABI nanoparticles were measured in aqueous solution (Figure 5). Since the PABI dye is not fluorescent, the encapsulation phenomenon could be checked by transmission measurements. The pure dye solution showed three typical absorption peaks characteristic of the aromatic macrocyclic π-electron of phthalocyanine dyes. Absorption maxima were recorded at 342 nm (B-band), 612 nm (vibrational band) and 668 nm (Q-band). The transmission spectra for the pure PABI and the samples 1a, 2a, 3a and 4a displayed almost the same profile in aqueous solution, though there was only a very slight red-shift (1-2 nm) for their absorbance maxima when compared to each PABI nanoparticles prepared respectively. Those results indicate that the four methods of encapsulation used were successful. The PABI dye seemed to be proper towards encapsulation conditions. Once embedded into the silica nanoparticles (samples 1a, 2a, 3a and 4a), the flat and rigid aromatic core of the phthalocyanine derivative can no longer escape, and remain well trapped within the silica network. Furthermore, phthalocyanine dyes are well-known to aggregate and generate π-stacking, and such phenomenon could emphasise the stability of those dyes towards encapsulation. The ordering of the π-stacking of the PABI molecules can favour their insertion into the silica network. Also, the π-stacking could be generated into the micelle, enhancing the rigidity of the organically bulk structure and therefore favouring the encapsulation process. Additionally, the interactions between the nitrogen atoms of the four imino bridges of the phthalocyanine aromatic core of the PABI, and the hanging hydroxyl of the silica core-shell facilitate further the encapsulation. The interactions of the dye to encapsulate with the silica network of the nanoparticles added to the rigidity of its aromatic core confer excellent conditions towards encapsulation. Prior to the results obtained with PABI, such conditions have been reported for the encapsulation of fluorescein 1 h and rhodamine B 1g. Similarly, those molecules possess reasonably flat and rigid aromatic cores, in part due to the conjugated system, emphasising the aromaticity and the stability of those dyes, and also due to the spiro centre contained in the structure of the fluorescein, and the lack of freedom towards the vertical bond in the molecule of rhodamine B, between the oxo-anthracenyl analogue core and the vertical ortho-carboxyphenyl substituent. The latest could introduce atropisomerism, exhibiting blocked isomers leading to rigid structures lacking of three-dimensional freedom, and therefore facilitating the encapsulation process.

#### 3.3.3. PPC porphyrin

**Figure 6:**
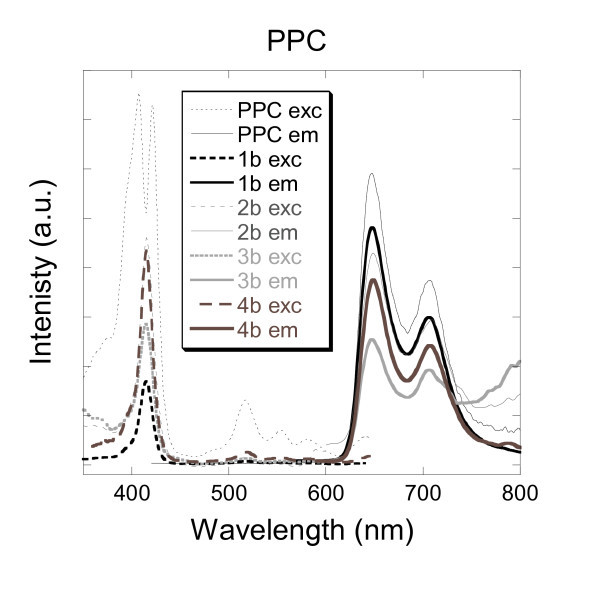
**Excitation and emission spectra of aqueous solutions of PPC and silica nanoparticles doped with PPC (1b, 2b, 3b and 4b)**.

Further incorporation of flat and rigid aromatic core organic dye has been investigated. The PPC porphyrin was chosen due to the structural similarity to the planar PABI molecule. But, exhibiting fluorescence, the PPC porphyrin was chosen to study the impact of encapsulation towards the fluorescent properties of this family of compounds. Indeed the PABI and the PPC molecules possess an aromatic core consisting of 18-π electrons, which emphasise the stability and the electrochromic properties of this family of intensely coloured dyes. The PPC dye exhibits fluorescence whereas the PABI detection was limited to absorption measurements. Excitation and emission spectra of a pure aqueous solution of PPC are illustrated in Figure 6.

The excitation spectrum displays a splitted maximum peak at 407 and 421 nm due to symmetry of the PPC molecule. Then the emission peaks were recorded at 647 and 706 nm. The fluorescence measurements of the silica-based samples 1b, 2b, 3b and 4b showed identical excitation and emission spectra than those exhibited by the free-PPC in water. As can be seen in Figure 6, the silica-based encapsulations showed a well-resolved coalesced peak for the excitation maxima at 415 nm. This phenomenon is typical of a loss of symmetry and of an ordered state of the organic molecules. This phenomenon could also be attributed to embedding stress which would result from the interaction of the organic dye with the silica matrix. This effect was observed for each process (1-4). The different encapsulation processes studied (1-4) did not alter whatsoever the emission spectra. As for the PABI experiments (1a, 2a, 3a and 4a), the successful encapsulation of PPC by mean of the four processes described earlier is a consequence of the flatness and rigidity of the aromatic macrocyclic core of the PPC porphyrin, as well as the possible interaction of the four nitrogens, of the residual pyrroles included in the porphyrin aromatic core, with the hanging hydroxyl substituents of the silica matrix.

#### IR 806

IR 806 is a water-soluble near-infrared cyanine dye. Usually these dyes are known to have narrow and intense absorption bands in the near-IR spectral region, and to possess good photostability. A solution of free IR 806 dye was used for fluorescence measurements in water.

**Figure 7:**
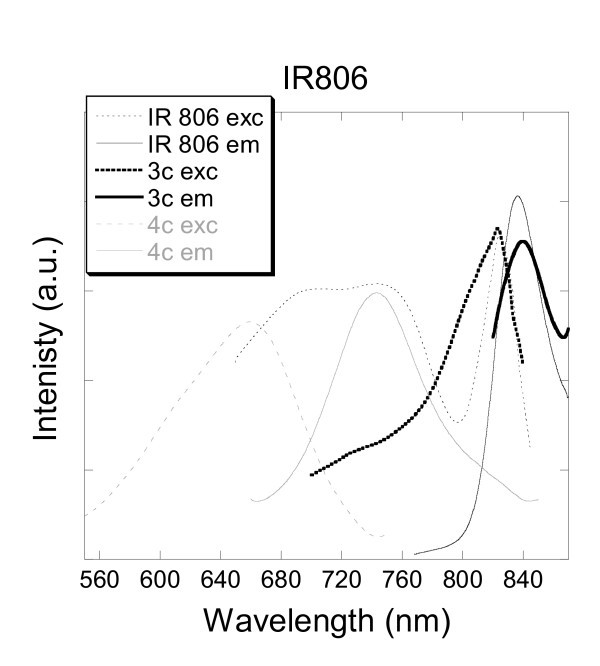
**Excitation and emission spectra of aqueous solutions of IR 806 and silica nanoparticles doped with IR 806 (3c, 4c)**.

The results are presented in Figure 7, and show three excitation peaks upon emission at 806 nm. The main excitation peak was observed as a sharp peak at 824 nm. Especially noteworthy was the observation of significant overlapping secondary peaks at 702 and 746 nm, equivalent in intensity. The emission peak was recorded at 837 nm. A comparison of the excitation and emission spectra measured for silica-based samples 1c, 2c, 3c and 4c gave various results. Fluorescence was measured but not recorded for samples 1c and 2c indicating the non-encapsulation of the IR 806 dye under those conditions. Most probably, the encapsulation's failures imply that the kinetic rate of hydrolysis of the TEOS prevent from ideal encapsulation conditions. Slow hydrolysis to produce the silica network can emphasise the exclusion of the molecule as well as an enhancement of the porosity of the silica network of the nanoparticles [55]. Hence, two straightforward explanations come to mind, either the dye is excluded during the growth of the silica matrix of the nanoparticle, or it is first encapsulated then released during the different washing steps due to the porosity of the silica network. Opposite results were observed for experiments 3c and 4c which encapsulations were successful. Fluorescent spectra of sample 3c are illustrated in Figure 5. The single excitation peak and emission peak were recorded at 827 and 839 nm, respectively. The slight bathochromic shift observed (2-3 nm) suggests an effect/influence of the confined IR 806 dye into the silica nanoparticles. Fluorescent spectra of sample 4c are also shown in Figure 7. Important hypsochromic shifts are observed as well as disappearance of the main sharp excitation peak occurring at 824 nm. The single excitation peak was recorded at 660 nm and the corresponding emission peak was observed at 743 nm. The encapsulation of IR 806 in the silica network of the nanoparticles tends to totally quench the low energy transition, therefore exhibiting only the secondary or high energy transition. Measurements of fluorescence of an aqueous solution of IR 806 did not exhibit luminescence at 743 nm upon excitation at 660 nm. The induced shift effect was observed and resulted from the confinement of the fluorescent dye within the silica particle, when prepared with TMOS. Subsequently, it is reasonable to assume that the interactions of the hydroxyl groups of the silica network with the IR 806 fluorescent dye tend to block preferably the radiative transitions at 806 nm than those at 743 nm. Furthermore, the successful encapsulation can result in the use of TMOS instead of TEOS which possess a faster rate of hydrolysis and build a denser silica network embedding more efficiently the IR 806 dye as explained in the paragraph above.

#### NBA

**Figure 8:**
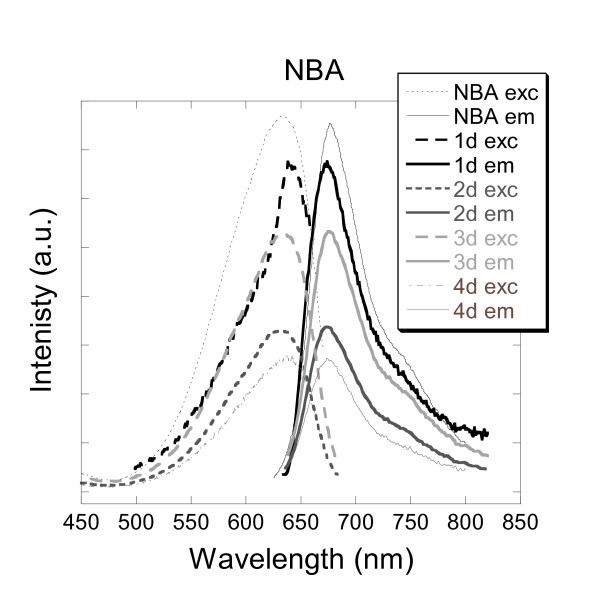
**Excitation and emission spectra of aqueous solutions of NBA and silica nanoparticles doped with NBA (1d, 2d, 3d and 4d)**.

The synthesis of nanosensors based on silica nanoparticles embedded with a rigid fluorophores called NBA was undertaken. NBA is commonly used as a fluorescent laser dye. An aqueous solution of free-NBA exhibited an excitation peak at 634 nm and an emission peak at 677 nm as illustrated in Figure 8.

Further attempts towards encapsulation of NBA using the four different methods detailed earlier on proved to be successful. Indeed reasonably similar maxima excitation and emission wavelengths were recorded in close range to those observed for the free-NBA. Subsequently, samples 1d, 2d, 3d and 4d gave excitation peaks at 641, 633, 633 and 637 nm, respectively, whereas the corresponding emission peaks were showed at 674, 674, 675 and 675 nm, respectively. The encapsulation tends to influence mostly the excitation peaks (Δ*λ*_ex _= 8 nm) than the emission peaks (Δ*λ*_em _= 3 nm). The water-soluble molecule of NBA dye was encapsulated successfully due in part to its rigid aromatic core. As for the PABI and PPC molecules, the rigidity of the aromatic core added to the presence of heteroatoms in the molecule of NBA tends to enhance the embedding process.

#### HITC and+ ICG

**Figure 9:**
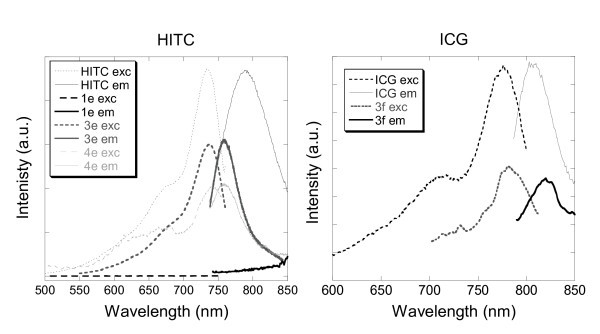
**Excitation and emission spectra of aqueous solutions of (left) HITC and silica nanoparticles doped with HITC 3e and 4e, and (right) ICG and silica nanoparticles doped with ICG 3f**.

Finally, two cyanine-based near-infrared absorbing dyes (HITC and ICG) were subjected to the four methods of encapsulations involved in this study. Those dyes are commercially available due to their photographic sensitivity and infrared lasers absorption, essential properties to the printing industry. It is also important to notice that, currently, the organic dye ICG is the only near-infrared fluorophores approved by FDA for use in vivo in humans [56]. Aqueous solutions of free-HITC and free-ICG displayed sharp excitation peaks at 734 and 776 nm, respectively, as well as sharp emission peaks at 790 and 806 nm as indicated in Figure 9.

Under encapsulation conditions of methods 1-4, HITC embedding occurred for samples 3e and 4e. Fluorescence was measured for 3e (*λ*_ex _= 738 nm, *λ*_em _= 758 nm) and 4e (*λ*_ex _= 741 nm, *λ*_em _= 759 nm), whereas no fluorescence could be recorded neither for samples 1e nor 2e. Furthermore, in the case of ICG, while sample 3f displayed a well-resolved fluorescence with an excitation peak at 780 nm and an emission peak at 820 nm, samples 1f, 2f and 4f did not exhibit any fluorescence. The poor chemical and photostability of cyanine-based dyes especially in aqueous environments under basic conditions, as well as their strong tendency to form aggregates might decrease their ability towards the encapsulation process (1-4). Also cyanine-based dyes must be monomolecular and possess planar rigid geometries to be efficient at absorbing and emitting light. Therefore, the poor rigidity of both cyanine-based molecules, HITC and ICG, indicates that it is a relevant criterion to take into account when proceeding to encapsulation of those dyes into silica nanoparticles. Samples 3e and 3f illustrated the successful encapsulation of HITC and ICG. This is in part due to the porosity of the silica nanoparticles, and also accentuated by the fact that those pores are functionalised with thiols (SH) that can bind and entrap organic dyes via hydrogen bondings and electrostatic forces.

## Conclusions

To conclude, these experiments have allowed us to establish and optimise criteria and principles towards efficient encapsulation of dyes by reverse microemulsion process involving non-covalent embeddement. Table 1 summarises the successful encapsulations as well as the techniques of characterisation used. The study of their luminescent properties or their quenching was also described.

**Table 1:** Reminder of successful and unsuccessful encapsulations of the different dyes (a-h) and the uses of the different methods of encapsulation (i.e. series 1-4) investigated during the study

Dyes		Series			
		1	2	3	4

a	PABI	✓	✓	✓	✓^a^
b	PPC	✓^a^	✓^a^	✓	✓^a^
c	IR 806	X^b^	X	✓	✓^b^
d	NBA	✓	✓^b^	✓	✓
e	HITC	X	X	✓^b^	✓
f	ICG	X	X	✓	X
g	RHOD B	✓^a^	N/A	N/A	N/A
h	FLUO	✓^a^	N/A	N/A	N/A

All successfully encapsulated samples have been further characterised by fluorescent measurements.

^a^Characterised by TEM analysis.

^b^Characterised by DLS measurements.

i. *Hydrophilic Vs hydrophobic character *the single use of TEOS allowed us to encapsulate hydrophilic molecules essentially. In order to embed molecules rather hydrophobic than hydrophilic into silica nanoparticles, the use of an additional silica precursor was considered to induce interactions of the silica with the selected dye via hydrogen bondings. Also, the choice of such silica precursors can be driven by their faster rates of hydrolysis to avoid steric exclusion.

ii. *Molecular rigidity (isomerism) *the rigidity of the dye has a propensity to favour the encapsulation process. The porosity and the density of the silica network of the so-formed nanoparticles are involved in an efficient encapsulation. Indeed, a denser silica matrix can generate rigid assembling molecules within the network, and therefore depending on the rate of hydrolysis of the chosen silica precursors.

iii. *Fluorescence display *the embeddement of a fluorescent dye does not prevent the display of fluorescence. Upon encapsulation, the dye-doped silica nanoparticles exhibit relatively similar excitation and emission spectra than the free dye. Interaction of the silica network and the chosen fluorescent dye should not cause quenching of its emission or influence the probability of some luminescent transitions between excited and ground states of the selected organic dye (as for IR 806). Blocking or shifting of the major radiative transition may be due to the interaction of the dye with the silica network or the locking of the isomerism. In couple of experiments, shifted peaks were observed, and those phenomena were attributed to interaction and/or affinity of the silica for the selected organic dye. Further interaction could give rise to total quenching of the fluorescent dye. Replacing or substituting the free hydroxyl groups of the silica by a different silica precursor during the synthesis has a tendency to modulate those interactions and to, sometimes, recover an emphasised emission signal from the selected fluorescent dye.

## Abbreviations

DLS: dynamic light scattering; HDTMOS: hexadecyltrimethoxysilane; MB: methylene blue; NBA: Nile Blue A perchlorate; PABI: propyl astra blue iodide; TEOS: tetraethoxysilane; TMOS: tetramethoxysilane; TBOS: tetra-*n*-butoxysilane; TEM: transmission electron microscopy; W/O: water in oil.

## Competing interests

The authors declare that they have no competing interests.

## Authors' contributions

The work presented here was carried out in collaboration between all authors. AA, OR and OP defined the research theme. AA and OR designed methods and experiments and also coordinated the present study. AA carried out the laboratory experiments, interpreted the results and wrote the paper. OR performed the luminescence measurements and analyzed the data. OP co-designed experiments and discussed analyses. JS: performed the syntheses of silica nanoparticles with rhodamine B and fluorescein dyes and the luminescence measurement of these samples. All authors have contributed to, seen, read and approved the manuscript.
